# 肺结节CT随访中的生长特性评估

**DOI:** 10.3779/j.issn.1009-3419.2017.08.14

**Published:** 2017-08-20

**Authors:** 大同 苏, 颖 王

**Affiliations:** 300052 天津，天津医科大学总医院放射科 Department of Radiology, Tianjin Medical University General Hospital, Tianjin 300052, China

**Keywords:** 肺结节, 计算机体层摄影, 随访, 生长模型, 倍增时间, Pulmonary nodule, Computed tomography, Follow-up, Growth model, Doubling time

## Abstract

对肺结节行计算机断层扫描（computed tomography, CT）随访并确定结节生长特性是临床针对不定性肺结节常采用的策略。依据肿瘤生长指数模型，常采用体积或质量倍增时间量化结节的生长速率。本文拟对肺癌的指数生长模型、肺结节生长量化评价的方法学、不同类型肺结节的生长特性进行综述。

## Abstract (en)

Computed tomography (CT) follow-up of indeterminate pulmonary nodules and the quantification of growth characteristics are the commonly adopted strategy in clinical setting. The volume/mass doubling time can be used to quantify the growth velocity based on exponential growth model. Therefore, we reviewed the followed aspects on growth evaluation of pulmonary nodules on chest CT, including the growth model of lung cancer, the methods used for nodule growth quantification and the growth characteristics of different types of pulmonary nodules.

肺结节的检出率随着计算机断层扫描（computed tomography, CT）技术的普及及发展明显增加^[1, 2]^。临床上偶然发现的肺小结节恶性率低，大多为良性病变。如何对偶然发现的肺内小结节进行评估和定性，一方面实现对恶性结节的早期诊断和治疗，另一方面避免对良性结节进行不必要的临床干预，目前仍是临床面对的难题。大量肺癌筛查研究应用CT对肺癌高危人群进行筛查，旨在检测出早期无症状肺癌病例，进而减低肺癌死亡率。这些研究在肺结节的处理方面积累了大量经验，其对肺癌风险度较低的结节的公认处理策略为CT随访观察，评估肺结节的生长特性。

结节的生长特性反映了结节内细胞数量或体积的增加与时间的关系。恶性结节中肿瘤细胞持续活跃的有丝分裂决定其常表现为持续快速的增长，而良性结节的生长速度则一般相对较慢^[3]^。量化恶性肺结节的生长速度不仅对鉴别良恶性结节具有重要的临床价值，还可以用来进一步规范肺结节随访CT的频率及间隔时间，例如对生长缓慢的磨玻璃密度结节的随访间隔可以延长，而对于生长迅速的实性结节随访间隔可以缩短。CT是目前对肺结节进行生长特性评估的主要方法，通过对系列CT中的结节进行测量可确定在一定时间内结节大小的变化。CT图像后处理技术的发展使结节三维体积测量成为现实，其较二维直径测量的准确度及重复性更佳。

本综述将对肺癌生长数学模型、肺结节生长特性评估的方法学及不同类型肺结节的生长特性分布进行总结。

## 1 肺结节定义

肺结节被Fleischner Society定义为：在胸片或CT扫描中圆形或椭圆形、不透明的、轮廓分明或不分明，测量直径 < 3 cm的由肺实质包围的肺内病灶^[4-7]^。根据高分辨率CT中实性成分所占结节的比重将肺结节分为3种不同的类型：纯磨玻璃密度（pure ground glass opacity, P-GGO）、混合磨玻璃密度（mixed ground glass opacity, MGGO）和实性结节（solid nodule）^[8]^，混合磨玻璃密度结节又称为部分实性结节（part-solid nodule），前两者又合称为亚实性结节。实性结节是指具有均匀的组织密度，掩盖了通过其内的支气管血管束；纯磨玻璃密度结节是指结节的密度较低，并不能掩盖其内的支气管血管束；而部分实性结节同时具有上述两个特征，最常见的形式是中心有实性密度，周围可见磨玻璃密度形成的晕^[9]^。

## 2 肺癌结节的指数生长模型

肿瘤的指数生长模型被广泛的用于评价包括肺癌在内的人类肿瘤的增长。指数生长模型最早是被Collins等^[10]^在1956年提出的。他们假设肿瘤起自于单个细胞，这个细胞经有丝分裂分裂为2个、4个、8个、16个……。假设忽略各种限制条件和环境因素，这个过程以稳定的速率持续下去，那么肿瘤就会呈指数的方式生长。最基本的指数方程可以被描述为：

*N*=2^*n*^

*N*是实际细胞的数量，*n*是细胞增殖的代数。倍增时间（double time, DT）可以被定义为肿瘤细胞数目增加一倍所需要的时间，还可以被定义为肿瘤体积增大一倍所需要的时间（volume doubling time, VDT），是最简单的描述肿瘤增长的一种方式。假设肿瘤的体积（Vt）与肿瘤细胞数量的增长呈正比，t是生长时间，当呈指数增长时，肿瘤的体积和时间的关系可以表述为：

Vt=V0×2^t/DT^

倍增时间可能依据体积（V）的直接测量来表达：

DTV=[In2×t]/[In(V2/V1)]

结节的体积如果依据结节的直径计算（D），假定肿瘤是一个球体，倍增时间可以用以下公式来计算：

DTD=[In2×t]/[3In(D2/D1)]

根据倍增模型，一个直径10 μm的细胞增长至一个直径1 mm的结节，大约需要20个倍增时间，再经过10个倍增时间可以长成直径1 cm的肿瘤，而后再经10个倍增时间，肿瘤直径会变成10 cm，重量可能达到1 kg。一般说来，每个肿瘤细胞的直径假设为25 μm，26个倍增周期可以产生一个直径1 cm的结节。这个模型表明一个肿瘤在它生命的大部分时期可能是小的或者生长缓慢的，直到它达到可诊断的大小，它才会生长非常迅速。

需要指出，指数模型是一种近似的模型，并不能完全预测肺癌的生长，其在临床中的应用主要在于评估其在一定时间内的生长速度。肺癌的生长是受多种因素调控。肿瘤细胞数目的增加主要是由3个基本参数决定：增殖细胞的细胞循环周期、细胞的增值率、细胞自发性衰亡的数量及比例^[11]^。肿瘤的体积也取决于3个因素：肿瘤细胞的大小；肿瘤细胞的数量；基质占肿瘤体积的比例、血液和其他非肿瘤因素。指数模型假设肿瘤体积与肿瘤细胞的数量呈正例，忽略基质，血液和大血管这些因素，并且假定肿瘤细胞的数量可以充分地反映肿瘤体积。事实上，整个肿瘤中肿瘤细胞的体积百分比可能因肿瘤类型而不同。例如，在早期肺癌中，半实性病变的肿瘤细胞的百分比可能与实性病变的不同。其次，肿瘤增长速度不仅取决于持续随机分裂，也取决于这些细胞与他们所处环境的关系。增长速度可能因为食物不足及空间不足或其他因素而在一定时期内出现变化^[12]^。肿瘤结节的出血可能造成肿瘤突然变大，相反地，主要供血血管可能随着坏死和自然缩小而形成血栓^[13]^。环境因素（如基本的营养、激素和化学因素）在某种情况下也会造成肿瘤增长速度的变化。再次，活跃性增长的肿瘤细胞的异质性可以影响指数模型的正确性。最后，肿瘤的增长不但与细胞增殖有关，并且和自发细胞衰亡的数量或比例有关^[11]^。

## 3 CT肺结节生长评价的方法学

肺结节生长速度量化的准确性主要取决于肿瘤体积量化的准确性。肺结节的测量包括传统的二维直径测量与计算机辅助体积定量。二维测量通常是测量结节的最大尺寸或者是肿瘤的最大径以及与其垂直的最大径的平均值作为结节的直径，然后应用球体或椭球体的计算公式计算其体积。结节的三维体积测量目前已经可以通过计算机实现。计算机软件根据结节与周围肺组织的CT值分布采用阈值分割及形态分析方法将结节完整分割，进而对其内的像素进行统计，通过像素的大小及数量将肺结节相关的像素转换成体积。

Yankelevitz等^[14]^对比了三维技术与二维技术在决定肿瘤体积之间的精确度。对于球型结节，三维体积测量和二维测量同样精确。对于不规则形状的结节，三维方法在评估肿瘤增长速度方面相较传统的肿瘤直径测量上更有优势。三维方法有在短时间内发现肿瘤大小的细微变化的潜力。例如，当一个3 mm的球形肿瘤倍增时，直径的增长百分比只有26.7%（3.8 mm）。通过测量直径来确定增长速度是非常困难的，但是体积却是增加了100%，检测起来就比较容易。

三维体积测量存在的问题是体积测量的重复性^[15]^。部分容积效应是结节体积测量的重要影响因素。基于阈值的方法常被用于将肿瘤从周围的肺实质中分离或者分割出来。软件将特定CT值（阈值）作为区分结节或者肺实质的标准。假设一个结节不能填充、占据整个像素，那么结节的CT值会与周围肺实质进行平均。如果一个体素的平均CT值高于阈值，那么就认为是肿瘤的一部分，但是如果扫描厚度增加导致平均CT值降低，那么就不能作为结节的一部分。因此，结节的总体积会随着区域厚度的不同而变化。Yankelevitz等^[14]^表明与1 mm层厚相比，0.5 mm的层厚的误差更小。Erasmus等^[16]^指出在CT扫描中肺肿瘤大小的测量经常是不一致的，这可能导致对肿瘤反应做出错误的解析。对于规则和不规则肿瘤来说，观察者间的差异均比观察者内大。荷兰比利时肺癌筛查实验应用的是三维体积测量^[17]^，11%的实性结节的三维测量重复性不佳，尤其是那些与支气管、胸膜相邻的结节，建议行人工校准^[18]^。应用体积测量使荷兰比利时肺癌筛查计划降低了假阳性病例的数量^[19]^。

非实性结节的评估要比实性结节复杂。非实性结节的生长不仅表现在体积增长，还表现在密度的变化^[20]^。在后续随访中，即使亚实性结节的直径稳定，但是实性部分的增加也往往提示可疑恶性结节^[21]^。针对这个问题，de Hoop等^[22]^介绍了一种估计肿瘤质量（用肿瘤的体积×结节的平均密度）的方法应用于非实性结节的测量。认为其灵敏性优于体积倍增时间。在其研究中，质量的计算是基于结节的体积及CT值，具体方法是通过体积定量测出体积，将结节的CT值加上1, 000作为密度，二者的乘积转化为质量。他们证明了通过这种质量检测方法可以检测结节早期的生长，并且相比于体积和直径测量更加灵敏可靠。总之，三维测量的优点在于其较二维测量更敏感，测量的准确度及重复性高，可适用于不对称结节。对亚实性结节，质量测量可能更能灵敏判断结节的生长。

## 4 不同类型肺结节的生长特性

### 4.1 结节生长的现有阳性标准

在肺癌早期干预计划研究中，结节增长的定义与结节的直径（结节的最大径和与其垂直的最大径的平均值）变化有关：对于 < 5 mm的结节，平均直径变化应≥50%，才认为是结节有增长；对于直径为5 mm-9 mm的结节，平均直径变化应≥30%；对于直径≥10 mm的结节，平均直径变化应≥20%^[23]^。而国家肺癌筛查实验（National Lung Screening Trial, NLST）定义结节的增长是认为直径增长超过10%^[24]^。在NELSON调查中，实性结节的增长被定义为：重复扫描间隔应至少3个月，结节的体积增长≥25%^[17]^。

### 4.2 肺结节的体积倍增时间

Friberg等^[25]^回顾了关于胸部X射线及CT的研究，并且对组织病理学进行定义，提出鳞状细胞癌和小细胞肺癌的增长速度明显高于腺癌。Hasegawa’s根据肺腺癌HRCT特点将肺腺癌分成了3类：磨玻璃密度，具有实性中心的磨玻璃密度和实性病变，通过对82例恶性结节进行3年的随访，得出了恶性实性结节的平均倍增时间为149 d、混合结节（亚实性结节）的平均倍增时间为457 d、磨玻璃密度结节的倍增时间为813 d^[26]^。Noguchi等^[27]^根据肿瘤生长模式对肺腺癌病理进行一个简单的组织学分类。他建议将腺癌分成6类。A类、B类和C类是以肺泡内皮细胞更换为生长模式的腺癌。A类肿瘤展现的增长模式是纯粹的肺泡内皮细胞置换，当肿瘤发展为B类的时候，肿瘤中出现坍塌的肺泡。当肿瘤发展成C类型的时候，肿瘤中由于促结缔组织增生性反应而出现中心纤维化，并且中心的纤维化会随着肿瘤的发展而变大。C类被认为是A类和B类更高级的阶段。D类、E类和F类型的腺癌的生长类型不同于ABC，是以堆积性生长方式为主的实性腺癌^[28]^。Takatoshi使用CT数据和组织病理学分类对周围型腺癌进行了评估^[29]^。在他们的研究中，6个A型或B型的腺癌有5个为磨玻璃密度，倍增时间的范围为662 d-1, 486 d，平均880 d。21个C型的腺癌中有15个表现为部分实性，倍增时为42 d-1, 346 d。D、E和F类型的7个肿瘤主要表现为实性密度，倍增时间为252 d-402 d，平均124 d。周围型肺腺癌主要以两种类型存在。第一种类型是在CT中表现为部分实性密度，并且生长速率较慢。另一种类型主要表现为实性的密度，但生长速度较快。因此，目前认为实性肺恶性结节的倍增时间常处于30 d-400 d^[30, 31]^。

对于良性结节，尽管生长机制有所不同，但其体积的增大也可以通过“倍增时间”来表现出来，目的是与恶性肿瘤进行对比。Diederich通过体积测量的方法计算出的恶性肺结节的倍增时间为20 d-400 d，生长过快或者生长过慢的结节往往预示着是良性的结节^[32]^。急性炎性病变在短时间内往往生长迅速，而慢性炎症或一些良性的结节的生长往往很缓慢^[33]^。

良性结节的另一诊断标准是1958年Good和Wilson提出，他们认为如果胸片上结节的稳定时间超过2年的话，那么可以认为这个病变是良性的^[34]^。自那以后，在医学界，连续观察2年结节稳定被广泛认为是良性结节的证据；2年的稳定性相当于至少730 d的倍增时间，这种说法使长倍增时间和明显的稳定性之间联系起来。稳定时间超过2年被认为是良性的观点仅适用于实性结节，这个观点不适用于亚实性结节，因为磨玻璃结节在表现出侵袭性之前可能会有一个很长的惰性生长期。

目前关于肺结节的倍增时间的研究大部分纳入的病例较少，关于结节的生长特性信息目前尚不全面，目前尚无公认的指南。

## 5 总结

肺结节的高发性及肺癌的致命性使结节的随访及生长评估在临床上十分普遍。体积测量并计算倍增时间对结节良恶性鉴别诊断具有较高价值。亚实性肺结节的质量测量兼顾体积与密度的变化，具有发展前景。不同密度的肺结节生长速率不同。目前尚无公认关于结节倍增时间的标准。
